# Metoclopramide-Induced Acute Dystonic Reactions May Be Associated With the CYP2D6 Poor Metabolizer Status and Pregnancy-Related Hormonal Changes

**DOI:** 10.3389/fphar.2019.00931

**Published:** 2019-08-22

**Authors:** Eng Wee Chua, Simon P. Harger, Martin A. Kennedy

**Affiliations:** ^1^Faculty of Pharmacy, Universiti Kebangsaan Malaysia, Kuala Lumpur, Malaysia; ^2^Hawke’s Bay District Health Board, Hastings, New Zealand; ^3^Carney Centre for Pharmacogenomics and Department of Pathology and Biomedical Science, University of Otago, Christchurch, Christchurch, New Zealand

**Keywords:** metoclopramide, acute dystonia, CYP2D6, poor metabolizer, pregnancy, estrogen, dopamine

## Abstract

We report two cases of metoclopramide-induced acute dystonia in pregnant women and consider the role of genetic variation in the pathogenesis of the adverse effect. By whole-gene sequencing, we found that both women were CYP2D6 poor metabolizers. We theorize that CYP2D6 governs the risk of metoclopramide-related acute dystonia through its role in the synthesis of serotonin, which inhibits the dopamine tone. The effect of CYP2D6 poor metabolism is exaggerated by rises in the estrogen levels during pregnancy, as the hormone augments dopamine sensitivity. Together, the two factors may create a hyper-dopaminergic state that is easily upset by metoclopramide, resulting in acute dystonia.

## Introduction

Metoclopramide is a commonly used antiemetic agent. It improves gut movement partly through its antagonistic action on dopamine, which has an inhibitory effect on the gut. However, because it disrupts central dopaminergic signaling, metoclopramide may produce rare movement disorders, such as acute dystonic reactions (Albibi and McCallum, 1983; Bateman et al., 1985). The incidence rate of metoclopramide-induced acute dystonia and akathisia has been reported as 0.5% (Bateman et al., 1989). To date, there is a dearth of studies that examined the genetic basis of metoclopramide-induced acute dystonia. Only one case report detailed the presence of the deleterious CYP2D6*4 allele in two patients who were given metoclopramide for chemotherapy-induced nausea (van der Padt et al., 2006). The allele involves a guanine-to-adenine transition within the intron 3-exon 4 boundary of *CYP2D6*, shifting the position of an “AG” acceptor site, illustrated as follows: 5′-CCACCCCCAGGACGCCCCTT-3′ to 5′-CCACCCCCAAGACGCCCCTT-3′. This causes erroneous splicing and deletion of one nucleotide from the fourth exon, thereby altering the ensuing reading frame. Consequently, a non-functional protein is produced (Hanioka et al., 1990).

It was thought that impaired CYP2D6 function reduced the clearance of metoclopramide and substantially increased the risk of acute dystonia caused by the drug (van der Padt et al., 2006). However, the assumption might have overlooked other factors that could also contribute to the development of the adverse effect. In the *CYP2D6*–metoclopramide case report, the chemotherapy that the patients received could have been equally culpable of the dystonic reactions. Chemotherapeutic agents have been reported to cause dystonia, possibly by adversely affecting the movement-related brain circuits, i.e., those housed in the basal ganglia (Brashear and Siemers, 1997).

In this work, we consider two cases of metoclopramide-triggered acute dystonia and the role of *CYP2D6* variation in the pathogenesis of the adverse effect. These cases were referred to us for genetic analysis, as part of a long-term initiative (Understanding Adverse Drug Reactions Using Genomic Sequencing, or UDRUGS) to systematically accrue cases of unusual adverse drug reactions in a biobank (Maggo et al., 2017). Based on the participants’ medical history and their *CYP2D6* and *SULT2A1* genotypes, we provide new insights into the relation between CYP2D6 poor metabolism, pregnancy-related hormonal changes, and acute dystonia caused by metoclopramide.

## Two Cases of Metoclopramide-Induced Acute Dystonia

### Case 1

Participant A, a 39-year-old female of European descent, was 12 weeks pregnant when the dystonic reaction occurred. She was taking oral metoclopramide 10 mg three times daily for pregnancy-associated hyperemesis, and not long thereafter (the exact interval was unclear), she started experiencing back spasms; however, she was not aware that metoclopramide was causing the adverse effect. After about 1 day of oral therapy, the vomiting persisted. She was given an additional 10-mg dose intravenously, and 3–4 h later, the back spasms developed into a full-blown dystonic reaction, resulting in torticollis and globally increased muscle tone. Also, she was unable to speak because her throat was “closing down.” She received treatment at a local hospital, and the reaction resolved after she was given three 0.5-mg intravenous doses of benztropine, which is an anticholinergic agent. On arrival at the hospital, her vital signs were normal. She was afebrile, with a heart rate of 80/min and an oxygen saturation level of 100%. Overall, we estimated that the gap between the first dose of metoclopramide and the occurrence of the acute dystonia was 1–2 days.

The participant consented to provide a blood sample for genetic analysis. We used a modified Pharmacist’s Workup of Drug Therapy form (Strand et al., 1988) to document her medical history. She was otherwise healthy except for a history of atopy. The only additional medication that she was taking was oral folic acid, which has no known interactions with metoclopramide. Using the Naranjo algorithm (Naranjo et al., 1981), we rated the causality of the adverse reaction as *possible*.

### Case 2

Participant B, a 32-year-old female of European descent, was 9–10 weeks pregnant when the dystonic reaction occurred. She was taking oral metoclopramide three times daily for pregnancy-associated nausea; the exact dosage was unknown. About 2 days after she started taking metoclopramide, she experienced the following symptoms: anxiety, jitteriness, involuntary upward movement of her eyes, neck spasms, and back arching. The symptoms subsided after she was given two doses of an “anti-Parkinsonian” agent. Because we had no access to the participant’s medical records, we did not have the details of her vital signs or other relevant physical examination findings for when she was treated for the adverse reaction.

The participant learned of UDRUGS by word of mouth, and she volunteered to take part in the project. We documented her medical history using the modified Pharmacist’s Workup of Drug Therapy form (Strand et al., 1988). She was healthy and had no underlying illnesses. The only medication that she was taking alongside metoclopramide was prenatal vitamins. The participant also described to us that she had previously taken metoclopramide at a lower dosage for a month (once instead of thrice daily), but she did not develop a reaction. Interestingly, she was not pregnant at the time. Using the Naranjo algorithm (Naranjo et al., 1981), we rated the adverse reaction as being *possibly* linked to metoclopramide.

## Genetic Analysis

We obtained blood samples from the two participants, extracted DNA, and Sanger-sequenced the *CYP2D6* and *SULT2A1* genes. The *CYP2D6* genotypes were determined using a previously reported method (Wright et al., 2010), whereby two duplex long PCRs were performed to detect *CYP2D6* deletion and duplication alleles and to amplify the entire gene (6.6 kb; **Supplementary Figure 1**). The co-amplification of a 3.5-kb product would indicate whole-gene deletion (*5) or duplication; but the product was absent from all the long PCRs performed for the participants. Therefore, we concluded that both participants did not harbor the duplication or the deletion allele. Using the diluted long amplicon as a template, we then performed touchdown PCRs to generate short, overlapping fragments that spanned *CYP2D6* and a portion of its promoter (**Supplementary Table 1**). We then Sanger-sequenced the resultant PCR products. We used a similar approach for *SULT2A1* sequencing and performed touchdown PCRs to generate products that covered the promoter region and all six exons of *SULT2A1* (**Supplementary Table 2**). The details of all the PCRs can be found in Supplementary Methods.

By whole-gene sequencing, we found that both participants were homozygous for the loss-of-function CYP2D6*4 allele (**Supplementary Figure 2**). In other words, the participants were CYP2D6 poor metabolizers. We did not identify any deleterious variants in their *SULT2A1* gene. Therefore, they were likely to have normal SULT2A1 function.

## Discussion

The acute dystonic reactions experienced by our participants were similar to those documented in other reports, namely, sustained contraction of the neck muscles, causing an arched back, and involuntary eye movements (Leus and van de Ven, 2015; Tianyi et al., 2017). Few studies have examined the genetic underpinnings of metoclopramide’s adverse effects (van der Padt et al., 2006; Parkman et al., 2012). To date, only *CYP2D6* variation has been linked to the risk of metoclopramide-related acute dystonia (van der Padt et al., 2006). It was believed that deficient CYP2D6 function increased the exposure to metoclopramide, though this was not confirmed by measurement of the drug levels (van der Padt et al., 2006). However, considering the current understanding of metoclopramide’s pharmacokinetics, we surmise that this is unlikely. Approximately 20% of metoclopramide is excreted unchanged, and 50% is renally eliminated in the conjugated form (Graffner et al., 1979). The major urinary metabolite is a sulfate conjugate, formed by SULT2A1. The de-ethylation and mono-oxygenation reactions, mediated largely by CYP2D6, are relatively minor and not believed to have a significant impact on plasma metoclopramide levels *in vivo* (Bateman et al., 1980; Desta et al., 2002; Senggunprai et al., 2009; Livezey et al., 2014). A previous study in children detected no relationship between metoclopramide levels and the occurrence of acute dystonia (Bateman et al., 1983).

Therefore, it seems more probable that the CYP2D6–metoclopramide relation is pharmacodynamic, not pharmacokinetic, in nature. CYP2D6 may regulate the central dopamine tone through its role in converting 5-methoxytryptamine to serotonin (Yu et al., 2003). Compared with the extensive metabolizers, the CYP2D6 poor metabolizers may have an inherently lower serotonin tone, allowing an unbridled increase in the dopamine tone. In a variety of studies, the interaction between the two neurotransmitters was thought to account for the association between CYP2D6 function and personality (Peñas-Lledó and Llerena, 2014). This suggests that variation in CYP2D6 activity is indeed accompanied by substantial biochemical changes in the brain.

**Figure 1:**
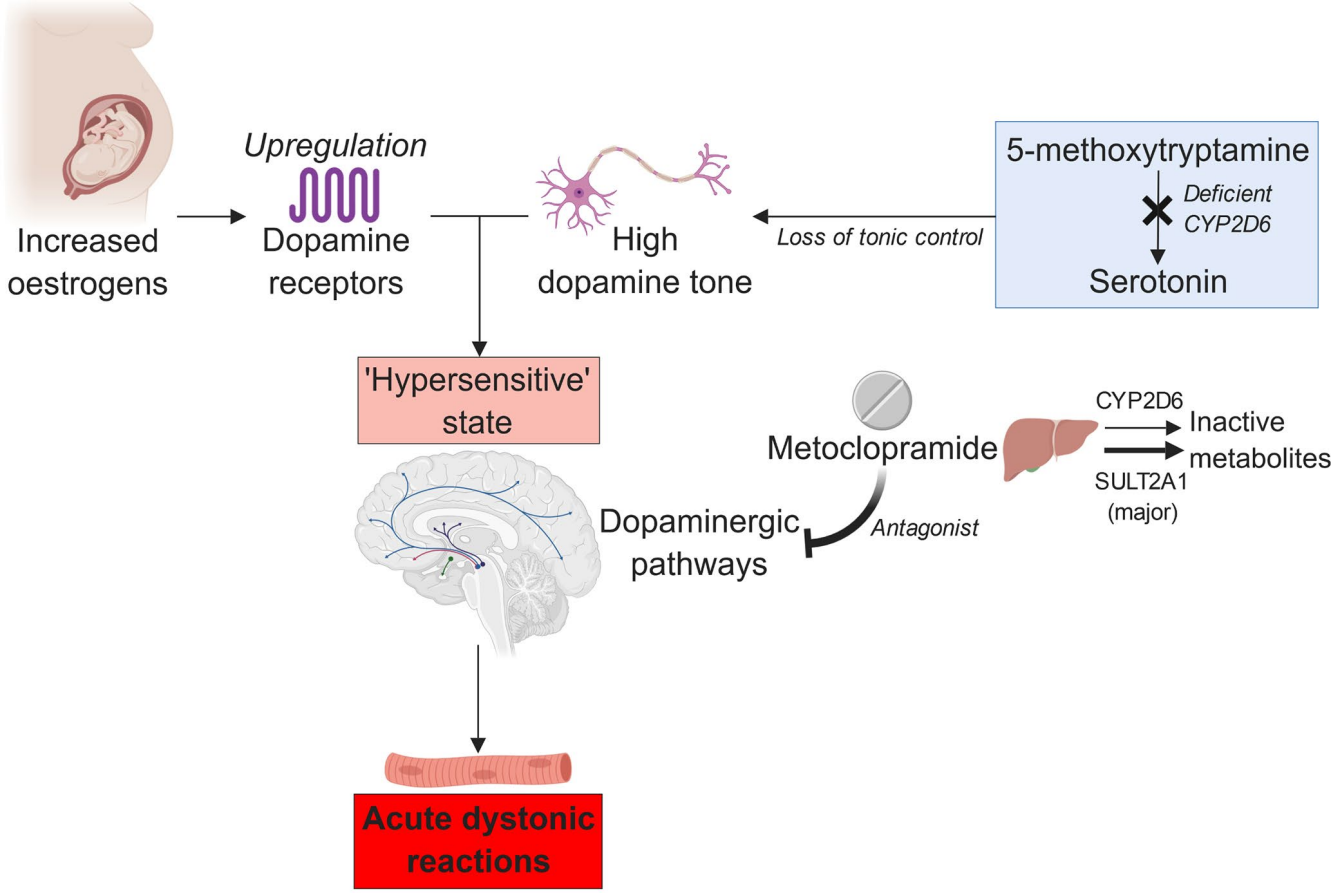
The proposed relation between estrogen, CYP2D6 poor metabolism, and metoclopramide-induced acute dystonia (created with BioRender).

However, impaired CYP2D6 function alone may not increase the dopamine tone sufficiently to trigger a major dystonic reaction in individuals taking metoclopramide. The influence of other physiological factors, such as sex hormone function, must also be present (Bateman et al., 1989). Otherwise, the incidence of the adverse effect would have been much higher, given the prevalence of the deleterious CYP2D6*4 allele among Caucasians; the reported allele frequency was ∼21% (Sachse et al., 1997). This hypothesis is supported by Case 2 participant’s reported tolerance of metoclopramide when she was not pregnant and the known association between oral contraceptive use and acute extrapyramidal reactions (Bateman et al., 1985). Estrogen can augment dopamine sensitivity by upregulating the dopamine receptors (Hruska and Silbergeld, 1980). Moreover, pregnancy-related physiological changes may decrease the CYP2D6 activity further in the poor metabolizers (Wadelius et al., 1997). Hence, we theorize that the combined influence of CYP2D6 poor metabolism and pregnancy-induced rises in the estrogen levels may create a delicate, hyper-dopaminergic state that is easily upset by metoclopramide (Askenasy et al., 1978), resulting in acute dystonia (**Figure 1**).

Nevertheless, it is worth noting that the presence of CYP2D6*4 in our participants could have been coincidental. In addition, the control of the number of dopamine receptors by estrogen appears to be a dynamic process. Administration of estradiol was shown in a study involving ovariectomized monkeys to prevent antipsychotic agents from inducing a compensatory increase in the dopamine receptors and causing “dopamine supersensitivity” (Bédard and Boucher, 1986).

## Conclusions

The two cases provide further evidence that the CYP2D6 poor metabolizer status may be associated with metoclopramide-induced dystonia and that the role of other mediating factors, including hormonal changes, warrants further investigation. Prescribing an alternative antiemetic agent to the CYP2D6 poor metabolizers could help to prevent the distressing adverse reaction. Future work may focus on assembling a cohort for confirming the association between *CYP2D6* variation and metoclopramide-induced dystonia. Measuring the blood concentrations of metoclopramide is necessary to ascertain the nature of the drug–*CYP2D6* interaction. It may also be worthwhile to investigate other factors that could drive the pathogenesis of metoclopramide-induced dystonia, such as age, gender, DNA variation in non-metabolic genes (e.g., dopamine receptor gene polymorphisms), and co-administered drugs. Finally, the cost-effectiveness of selectively prescribing metoclopramide based on *CYP2D6* genotypes should be determined.

## Data Availability

The raw data supporting the conclusions of this manuscript will be made available by the authors, without undue reservation, to any qualified researcher.

## Ethics Statement

The project was approved by the Southern Health and Disability Ethics Committee (New Zealand). Written consent to take part in this study was obtained from the participants by face-to-face interviews. They understood that the result of their genetic analysis, alongside their clinical data, might be published in a journal article; however, their identity would remain confidential.

## Author Contributions

EWC and MK recruited the participants. EWC performed the experiments and wrote the manuscript. MK and SH reviewed the initial and final drafts of the manuscript.

## Conflict of Interest Statement

EC and MK received funding from the University of Otago, Christchurch, for the submitted work; there were no financial relationships with any organizations that might have an interest in the submitted work in the previous 3 years; and there were no other relationships or activities that could appear to have influenced the submitted work.

The remaining author declares that the research was conducted in the absence of any commercial or financial relationships that could be constructed as a potential conflict of interest.
