# Supplementary material for: Metoclopramide-Induced Acute Dystonic Reactions May Be Associated With the CYP2D6 Poor Metabolizer Status and Pregnancy-Related Hormonal Changes
Source: Front Pharmacol. 2019 Aug 22;10:931. doi: 10.3389/fphar.2019.00931 (PMC6713716; doi:10.3389/fphar.2019.00931)
Supplement: Supplementary file 1 [file DataSheet_1.pdf]

SUPPLEMENTARY METHODS

PCR set-up

All PCRs were set up in a separate area or in a PCR-preparation room. The equipment used for reaction set-up, including pipettes, pipette tips, microfuge tubes, and strip tubes, were pre-irradiated with UV light for about 15-20 min to minimise the risk of carry-over contamination. The reaction mixtures were kept at 4°C prior to thermal cycling to minimise the unspecific amplification that may arise from residual *Taq* DNA polymerase activity at low temperatures.

CYP2D6 genotyping and Sanger sequencing

Two duplex long-range PCRs were used to screen for *CYP2D6* whole-gene deletion or duplication alleles that arise from recombination events at the *CYP2D* gene locus. The cross-over occurs within the repetitive elements adjacent to the *CYP2D* genes, thereby creating unique downstream sequences that can be distinguished by specific primers.

A 10 µL PCR for the detection of *CYP2D6* duplication allele(s) was set up as follows: 1× KAPA™ LongRange Reaction Buffer, 1.75 mM Mg^{2+}, 0.3 mM of each dNTP, 0.4 µM each of 6.6kb-F and 6.6kb-R, 0.3 µM each of 2D6 Dup Frag B F and 2D6 Dup Frag B R, 1 M betaine, 0.25 U of KAPA™ LongRange DNA Polymerase (Kapa Biosystems, Inc., Wilmington, MA, USA), and 50 ng of DNA. The reaction mixture was subjected to initial heating at 94°C for 3 min, followed by 35 cycles of 94°C for 25 s, 68°C for 10 s, and 68°C for 7 min, and a final elongation step of 72°C for 7 min. Another PCR for the detection of *CYP2D6* deletion allele(s) was similarly set up, except that the primers 2D6 Dup Frag B F and 2D6 Dup Frag B R were replaced by CYP-13 and CYP-24.

The primers 6.6kb-F and 6.6kb-R amplified the entire *CYP2D6* gene plus a portion of the intergenic region, generating a 6.6-kb PCR product (Fragment A; Supplementary Figure 1). The presence of the duplication or deletion allele(s) would cause additional amplification of a 3.5-kb product (Fragment B or C). Subsequently, the long PCR product was diluted 1000-fold, and 1 µL of the diluted products was used as the templates in nine nested PCRs to prepare short amplicons for Sanger sequencing (Supplementary Table 1). The PCR mixtures comprised 1× reaction buffer, 1 µL of the diluted long amplicon, 0.2 mM of each dNTP, 0.2 µM each of the forward and reverse primers, 1.5 mM Mg^{2+}, and 0.025 U/µL of TAQ-TI Heat-Activated DNA Polymerase (Fisher Biotec, Wembley, WA, Australia). A stringent touchdown PCR protocol was used with a starting temperature of 70°C, which was successively decreased by 1°C per cycle to reach a target temperature of 55°C.

Supplementary Table 1. Primers used for *CYP2D6* genotyping and Sanger sequencing.

**Primer name**	**Sequence (5'-3')**	**Product length**	**Reference**
Long PCR (the entire CYP2D6 gene)			
6.6kb-F	ATG GCA GCT GCC ATA CAA TCC ACC TG	6.6 kb	Gaedigk *et al.* 2007
6.6kb-R	CGA CTG AGC CCT GGG AGG TAG GTA G		Gaedigk *et al.* 2007
Duplex long PCR (whole-gene deletion)			
CYP-13	ACC GGG CAC CTG TAC TCC TCA	3.5 kb	Steen *et al.* 1995
CYP-24	GCA TGA GCT AAG GCA CCC AGA C		Steen *et al.* 1995
Duplex long PCR (whole-gene duplication)			
2D6 Dup FragB F	CCA TGG AAG CCC AGG ACT GAG C	3.5 kb	Gaedigk *et al.* 2007
2D6 Dup FragB R	CGG CAG TGG TCA GCT AAT GAC		Gaedigk *et al.* 2007

Cont'd: Supplementary Table 1

Primer name	Sequence (5'-3')	Product length	Reference
CYP2D6 sequencing			
6.6kb-F	ATG GCA GCT GCC ATA CAA TCC ACC TG	1071 bp	Gaedigk *et al.* 2007
2D6-1496/2SR	CCA GTC ACA CAC ACA TAC AGA CCC GGC		NA
2D6PromF	TCC TCC ATA ACG TTC CCA CCA GAT	1071 bp	Wright *et al.* 2010
2D6PromR	CCA TAC CTG CCT CAC TAC CAA ATG		Wright *et al.* 2010
2D6Ex1F	TCT GGA GCA GCC CAT ACC CG	1062 bp	Wright *et al.* 2010
2D6Ex1SR	CCC CAG ACT ACA GGT CCT AGT CCT ATT TG		NA
2D6Ex2F	TCC TCC TTC CAC CTG CTC AC	675 bp	Wright *et al.* 2010
2D6Ex2SR	CTT TGC CCC ACC TCG TCT CT		NA
2D6Ex34F	AGC TGG AAT CCG GTG TCG AA	1070 bp	Wright *et al.* 2010
2D6Ex34SR	AGC CAT CTC CAG GTA GAC CCA G		NA
2D6Ex56F	ACA GGC AGG CCC TGG GTC TA	884 bp	Wright *et al.* 2010
2D6Ex56SR	CCT GGT CAC CCA TCT CTG GTC		NA
2D6Ex7F	CCA ACA TAG GAG GCA AGA AG	691 bp	Wright *et al.* 2010
2D6Ex7R	ACT GGA CTC TAG GAT GCT GG		Wright *et al.* 2010
2D6Ex8F	CAG AAT GTT GGA GGA CCC AA	612 bp	Wright *et al.* 2010
2D6Ex8R	AGG AAA GCA AAG ACA CCA TG		Wright *et al.* 2010
2D6Ex9F	TGA AGG ATG AGG CCG TCT GG	920 bp	Wright *et al.* 2010
2D6-R	ACT GAG CCC TGG GAG GTA GGT AG		Gaedigk and Coetsee 2008

SULT2A1 PCRs and sequencing

Seven amplicons covering the promoter region and all six exons of the *SULT2A1* gene were generated using the primers listed in Supplementary Table 2. The PCR mixtures comprised 1× reaction buffer, 2.5 ng/µL of template DNA, 0.2 mM of each dNTP, 0.2 µM each of the forward and reverse primers, 1.5 mM Mg^{2+}, and 0.025 U/µL of HOT FIREPol® DNA Polymerase (Solis BioDyne, Tartu, Estonia). The final concentrations of all primers were 0.3 µM. A touchdown protocol was used to run all reactions simultaneously. The PCR was initiated with an annealing temperature of 65°C, which was decreased by 1°C per cycle to reach a target temperature of 50°C.

Supplementary Table 2. Primers used for *SULT2A1* sequencing.

Primer name	Sequence (5'-3')	Product length	Reference
SULT2A1UpsF	AAG ATG AGA ACA GAT AAA GAC TGT G	537 bp	NA
SULT2A1UpsR	CGA CAT GAT GAT GAC CTC TT		NA
SULT2A1Exn1F	TAA ACT TTA CAA CAA ACA TGT GAC	431 bp	NA
SULT2A1Exn1R	TTC TCT GAT TGT CAA TGG TAT TAG G		NA
SULT2A1Ex2F	ATG TCC GGC TGA GAT GGT ACA	372 bp	Thomae *et al.* 2002
SULT2A1Ex2R	AAC ACA GAC CTG TTG AAG GAG		Thomae *et al.* 2002
SULT2A1Exn3F	AAA TTT GAT TAG TGA GTG CAG TAA G	455 bp	NA
SULT2A1Exn3R	GCT AGA GTT CTG CTG CTA TG		NA
SULT2A1Exn4F	CTG ACT AAT ACA CTG TCA TTC CAT A	456 bp	NA
SULT2A1Exn4R	TTA ATC CTG CTC TTT GTG ACT CTT C		NA
SULT2A1Ex5F	GGG ATT ACA GGC GTG AAC CAC C	351 bp	Thomae *et al.* 2002
SULT2A1Ex5R	GCA CTC TTT CAT CTC AAC TGT T		Thomae *et al.* 2002
SULT2A1Exn6F	GAT AAA GGC CCA CCA CGA AC	367 bp	NA
SULT2A1Exn6R	GCA GAG GTT TGA TAT TTA AGG TTT		NA

SUPPLEMENTARY FIGURES

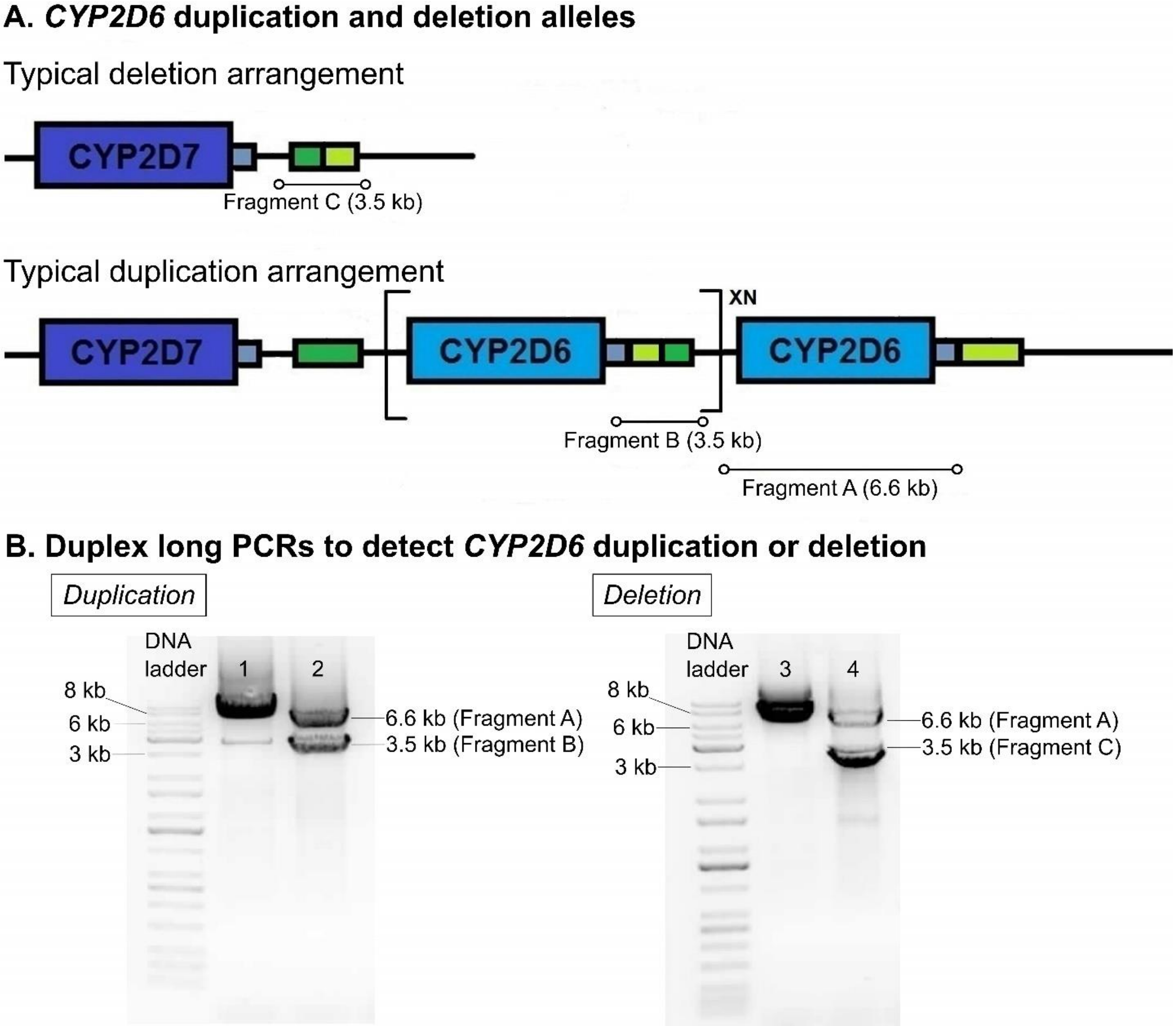

Supplementary Figure 1. **A.** Typical *CYP2D6* gene structures following recombination events that result in whole-gene deletion or duplication. The attendant, chimeric sequences downstream of *CYP2D6* and *CYP2D7* (represented by the two-coloured blocks) are formed by exchange of gene segments between the two genes and can be detected by specific primers, yielding a 3.5 kb product (Gaedigk *et al.* 2007). **B.** Duplex long PCRs performed to detect the *CYP2D6* duplication or deletion allele(s) using the DNA sample from the Case 2 participant (lanes 1 and 3). A sample with known *CYP2D6* duplication and deletion alleles was used as a positive control (lanes 2 and 4). Negligible or no amplification of the 3.5 kb product indicated the absence of the duplication or deletion allele(s). Though not shown in the gel image, a no-template control was also included in each PCR to check for cross-contamination.

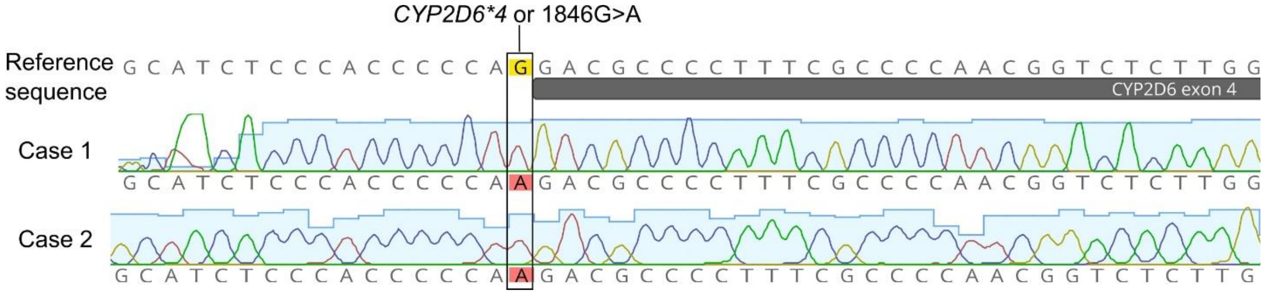

Supplementary Figure 2. Sanger sequences, aligned against M33388.1 (*CYP2D6*), showing the presence of the loss-of-function *CYP2D6*4* in both participants.

REFERENCES

Gaedigk A, Ndjountché L, Divakaran K *et al*. Cytochrome P4502D6 (*CYP2D6*) gene locus heterogeneity: characterization of gene duplication events. *Clin. Pharmacol. Ther*. 81(2), 242-251 (2007).

Gaedigk A, Coetsee C. The *CYP2D6* gene locus in South African Coloureds: unique allele distributions, novel alleles and gene arrangements. *Eur. J. Clin. Pharmacol.* 64(5), 465-475 (2008).

Gaedigk A, Jaime LK, Bertino JS Jr *et al*. Identification of novel *CYP2D7-2D6* hybrids: non-functional and functional variants. *Front. Pharmacol.* 1, 121 (2010).

Steen VM, Molven A, Aarskog NK, Gulbrandsen AK. Homologous unequal cross-over involving a 2.8 kb direct repeat as a mechanism for the generation of allelic variants of human cytochrome P450 CYP2D6 gene. *Hum. Mol. Genet.* 4(12), 2251-2257 (1995).

Thomae BA, Eckloff BW, Freimuth RR, Wieben ED, Weinshilboum RM. Human sulfotransferase SULT2A1 pharmacogenetics: genotype-to-phenotype studies. *Pharmacogenomics J.* 2(1), 48-56 (2002).

Wright GEB, Niehaus DJH, Drögemöller BI, Koen L, Gaedigk A, Warnich L. Elucidation of *CYP2D6* genetic diversity in a unique African population: implications for the future application of pharmacogenetics in the Xhosa population. *Ann. Hum. Genet.* 74(4), 340-350 (2010).
